# Efficacy of immune checkpoint inhibitor therapy for advanced urothelial carcinoma in real-life clinical practice: results of a multicentric, retrospective study

**DOI:** 10.1038/s41598-023-44103-9

**Published:** 2023-10-13

**Authors:** Melinda Váradi, Orsolya Horváth, Orsolya Módos, Tamás Fazekas, Camilla M. Grunewald, Günter Niegisch, Ulrich Krafft, Viktor Grünwald, Boris Hadaschik, Csilla Olah, Anikó Maráz, Andrea Furka, Miklós Szűcs, Péter Nyirády, Tibor Szarvas

**Affiliations:** 1 Department of Urology, Semmelweis University, Üllői út 78/B, Budapest, 1082 Hungary; 2 Department of Genitourinary Medical Oncology and Pharmacology, National Institute of Oncology, Budapest, Hungary; 3 Department of Urology, Medical Faculty and University Hospital Duesseldorf, Heinrich-Heine-University Duesseldorf, Duesseldorf, Germany; 4 Department of Urology, University of Duisburg-Essen, Hufelandstr 55, 45147 Essen, Germany; 5 Department of Oncotherapy, University of Szeged, Szeged, Hungary; 6 Department of Oncology, Faculty of Medicine, University of Debrecen, Debrecen, Hungary; 7 Department of Clinical Radiology, Institute of Practical Methodology and Diagnostics, Faculty of Health Care, University of Miskolc, Miskolc, Hungary

**Keywords:** Cancer, Drug discovery, Immunology, Diseases, Oncology, Risk factors, Urology

## Abstract

Clinical trials revealed significant antitumor activity for immune checkpoint inhibitors (ICI) in metastatic urothelial carcinoma (mUC). Due to their strict eligibility criteria, clinical trials include selected patient cohorts, and thus do not necessarily represent real-world population outcomes. In this multicentric, retrospective study, we investigated real-world data to assess the effectiveness of pembrolizumab and atezolizumab and to evaluate the prognostic value of routinely available clinicopathological and laboratory parameters. Clinical and follow-up data from mUC patients who received ICIs (01/2017-12/2021) were evaluated. Overall survival (OS), progression-free survival (PFS), objective response rate (ORR), disease control rate (DCR), and duration of response (DOR) were used as endpoints. Patients’ (n = 210, n = 76 atezolizumab and 134 pembrolizumab) median OS and PFS were 13.6 and 5.9 months, respectively. Impaired ECOG-PS, the presence of visceral, liver or bone metastases, and hemoglobin levels were independently associated with poor OS and DCR. Furthermore, Bellmunt risk factors and the enhanced Bellmunt-CRP score were shown to be prognostic for OS, PFS and DCR. In conclusion, ICIs are effective treatments for a broad range of mUC patients. Our results confirmed the prognostic value of numerous risk factors and showed that Bellmunt risk scores can further be improved when adding CRP to the model.

## Introduction

Platinum-based chemotherapy has been the standard treatment for patients with locally advanced or metastatic urothelial carcinoma (mUC) for over 3 decades. In the past few years, the therapeutic landscape of locally advanced or mUC has remarkably changed, as immune checkpoint inhibitors (ICI) targeting programmed cell death protein-1 (PD-1) or programmed cell death-ligand 1 (PD-L1) have been approved for the treatment of mUC^1^. Data from the two-cohort, single-arm, multicenter phase II IMvigor210 trial have led to accelerated approval of the first ICI, atezolizumab, a monoclonal antibody directed against PD-L1 at first in the platinum-refractory, and later also in the first-line setting^2^.

Subsequently, four other inhibitors have been approved (avelumab, durvalumab, nivolumab, pembrolizumab) in the cisplatin refractory setting^3–6^. The anti-PD-1 antibody, pembrolizumab proved to be superior in terms of overall response rate (ORR), complete response (CR), and partial response (PR) and showed overall survival (OS) benefit of 3 months compared to second-line chemotherapy in a multicenter, randomized, active-controlled phase III trial (KEYNOTE-045)^6,7^. Later pembrolizumab and atezolizumab had also been approved in the first-line setting^8^, however due to the early results of two clinical trials (KEYNOTE-361 and IMvigor130) the approval has been restricted to patients with high.

PD-L1 tissue expression^9^. The role of PD-L1 expression as a biomarker for the selection of patients who are more likely to benefit from ICI therapy remains controversial. It was shown to be associated with better response rates to second-line atezolizumab (9% vs. 26%; IMvigor 210 study) and to first-line pembrolizumab treatments (20% vs. 38%; KEYNOTE-052)^2,8^. According to this later results, a recent meta-analysis revealed a survival benefit for PD-L1–positive mUC patients receiving first-line ICI treatment compared to those receiving standard chemotherapy^10^. However, the negative predictive value of low PD-L1 expression should rather be considered as low, as pembrolizumab improved survival also in the PD-L1 low expression subgroup as shown in the KEYNOTE-045 study^7^. In addition, to ICI therapy, novel treatment options such as targeted anti-FGFR therapy or antibody–drug conjugates became available and providing further therapeutic options for mUC patients^11^.

The above-mentioned clinical ICI-trials have included strongly selected patient populations and have been conducted under standardized and strictly controlled conditions in order to minimize bias and potential confounders^12^. Since the populations enrolled in such studies may differ in many ways (age, comorbidities, generalized organ dysfunction) from those seen in everyday practice, study findings are not always generalizable to all patient populations^13^. Therefore, there is a need for real-world data to provide information on the efficacy of ICI treatment in the general population.

In this multicentric, retrospective study we examined real-world data in order (1) to assess the characteristics of ICI-treated urothelial cancer (UC) patients, (2) to examine the effectiveness of two widely used ICI agents (pembrolizumab and atezolizumab) comparing to respective randomized controlled trials (RCTs) and (3) to evaluate the prognostic value of routinely available clinicopathological and laboratory parameters.

## Results

### Patient characteristics

Data from 210 eligible patients were analyzed. A full description of patient characteristics for the whole cohort as well as for the first- and second-line treated patients are shown in Table 1. Seventy-six patients received atezolizumab and 134 patients received pembrolizumab. The median age at therapy initiation was 67.3 years (range: 28.9–87.2). Bellmunt risk calculation was possible for 184 patients^14^ (Table 1A).

**Table 1:** Patients, treatment (A) and follow-up (B) characteristics for the whole cohort and by treatment lines.

A			
Variables	Whole cohort	First-line	Second-line
	n (%)	n (%)	n (%)
Total number of patients	210	84	126
Age at diagnosis, years median (range)	67.3 (28.9–87.2)	70.0 (45.2–87.2)	65.3 (28.9–86.3)
Age at ICI initiation, years median (range)	69.8 (29.4–88.8)	71.9 (45.3–88.4)	68.2 (29.4–88.8)
Sex			
Male	146 (69.5)	51 (60.7)	95 (75.4)
Female	64 (30.5)	33 (39.3)	31 (24.6)
Location of primary tumor			
UTUC	26 (12.4)	6 (7.1)	20 (15.9)
BC	172 (81.9)	74 (88.1)	98 (77.8)
Both	12 (5.7)	4 (4.8)	8 (6.3)
Setting			
1L	84 (40.0)	84 (100)	–
2L	126 (60.0)	–	126 (100.0)
Drug			
Atezolizumab	76 (36.2)	18 (21.4)	58 (46.0)
Pembrolizumab	134 (63.8)	66 (78.6)	68 (54.0)
Prior treatments			
NAC	20 (9.5)	7 (8.3)	13 (10.3)
RCE	63 (30.0)	22 (26.2)	41 (32.5)
RNU	28 (13.3)	8 (9.5)	20 (15.9)
CTX (IC/AC/PC)	126 (60.0)	–	126 (100.0)
RCT	22 (10.5)	18 (21.4)	4 (3.2)
RT	77 (36.7)	38 (45.2)	39 (31.0)
ECOG-PS at ICI initiation			
0	117 (55.7)	39 (46.4)	78 (61.9)
1	58 (27.6)	28 (33.3)	30 (23.8)
2	22 (10.5)	15 (17.9)	7 (5.6)
3	2 (1.0)	–	2 (1.6)
Unknown	11 (5.2)	2 (2.4)	9 (7.1)
Metastatic sites			
LN	144 (68.6)	55 (65.5)	89 (70.6)
Only LN	67 (31.9)	29 (34.5)	38 (30.2)
Liver	33 (15.7)	9 (10.7)	24 (19.0)
Visceral	95 (45.2)	32 (38.1)	63 (50.0)
Bone	49 (23.3)	9 (10.7)	40 (31.7)
No metastases	22 (10.5)	16 (19.0)	6 (4.8)
Bellmunt risk factors			
0	74 (35.2)	19 (22.6)	55 (43.7)
1	81 (38.6)	43 (51.2)	38 (30.2)
2	24 (11.4)	9 (10.7)	15 (11.9)
3	5 (2.4)	–	5 (4.0)
Unknown	26 (12.4)	13 (15.5)	13 (10.3)
Bellmunt-CRP			
0	33 (15.7)	10 (11.9)	23 (18.3)
1	38 (18.1)	23 (27.4)	15 (11.9)
2	15 (7.1)	5 (6.0)	10 (7.9)
3 +	7 (3.3)	1 (1.2)	6 (4.8)
Unknown	117 (55.7)	45(53.6)	72 (57.1)

B			
Variables	Whole cohort	First-line	Second-line
	n (%)	n (%)	n (%)
Time of follow-up, months, median (range)	10.2 (0–68.7)	10.2 (0.5–68.7)	10.2 (0–64.8)
Number of cycles of ICI, median	6 (1–80)	7 (1–47)	5 (1–80)
ICI treatment (months), median (range)	4.3 (0–54.6)	4.95 (0–31.8)	3.6 (0–54.6)
ICI treatment ongoing at last follow-up	31 (14.8)	13 (15.5)	18 (14.3)
Best overall response			
Complete response	13 (7.2)	8 (11.0)	5 (4.6)
Partial response	53 (29.3)	16 (21.9)	37 (34.3)
Stable disease	46 (25.4)	24 (32.9)	22 (20.4)
Progressive disease	69 (38.1)	25 (34.2)	44 (40.7)
No radiologic evaluation performed	29	11	18
ORR	66 (36.5)	24 ( 32.9)	42 (38.9)
DCR	112 (61.9)	48 (65.8)	64 (59.3)
DOR, months, median (range)	11.8 (0.1–67.7)	11.4 (0.4–67.7)	11.9 (0.1–62.9)
PFS, months, median (95% CI)	5.9 (3.9–7.8)	7.2 (4.2–10.3)	4.4 (2.3–6.5)
Death at last follow-up	140 (66.7)	50 (59.5)	90 (71.4)
OS, months, median (95% CI)	13.6 (9.4–17.7)	13.7 (10.0–17.5)	13.6 (7.2–19.9)

*UTUC* upper tract urothelial carcinoma, *BC* bladder cancer, *1L* first-line, *2L* second-line, *NAC* neoadjuvant chemotherapy, *RCE* radical cystectomy, *RNU* radical nephroureterectomy, *CTX* chemotherapy, *IC* induction chemotherapy, *AC* adjuvant chemotherapy, *PC* palliative chemotherapy, *RCT* radio chemotherapy, *RT* radiotherapy, *ECOG-PS* Eastern Cooperative Oncology Group performance status, *LN* lymph node, *ORR* overall response rate, *DCR* disease control rate, *DOR* duration of response, *PFS* progression-free survival, *OS* overall survival, *CI* confidence interval.

### Efficacy (tumor responses, PFS, OS)

Patients received a median of 6 (range: 1–80) treatment cycles and remained on therapy for a median of 4.3 months (range: 0–54.6). At the time of the data cutoff, 31 patients (14.8%) were still receiving ICI drugs. The median follow-up period after ICI initiation was 10.2 months. Among the 181 patients evaluable for tumor response, 13 had CR and 53 had PR. ORR for the whole cohort was 36.5%, while for the first-line 32.9% and for the second-line setting 38.9%. The median DOR was 11.8 months. Disease control was achieved in 112 patients.

The median PFS for the whole cohort and for the first-line and the second-line settings were 5.9 months (95% CI 3.9–7.8), 7.2 months (95% CI 4.2–10.3) and 4.4 months (95% CI 2.3–6.5), respectively. A total of 140 patients (66.7%) died during the follow-up period. The median OS was 13.6 months (95% CI 9.4–17.7) (Table 1B).

### Factors associated with OS and PFS

In univariate Cox regression analyses, prior radical surgery, LN (lymph node) only metastases, high baseline hemoglobin and albumin as well as high eGFR values were significantly associated with improved OS. The presence of liver, visceral or bone metastases, worse ECOG-PS, presence of any Bellmunt risk factor (1 +) Bellmunt-CRP risk factor (1 +), and elevated NLR values were associated with shorter OS (Table 2).

**Table 2:** Univariate Cox regression analysis.

Variables		Overall survival				Progression-free survival			
		n	HR	95% CI	p	n	HR	95% CI	p
Age at ICI initiation	≤ 68	88	Ref			88	Ref		
	> 68	122	0.907	0.649–1.269	0.569	122	0.711	0.521–0.970	**0.032**
Sex	Male	146	Ref			146	Ref		
	Female	64	1.071	0.744–1.539	0.713	64	0.952	0.677–1.340	0.779
Tumor site	Bladder	172	Ref			172	Ref		
	Upper urinary tract	26	0.733	0.428–1.255	0.258	26	0.904	0.565–1.448	0.675
ICI drug	Atezolizumab	76	Ref			76	Ref		
	Pembrolizumab	134	0.905	0.645–1.270	0.563	134	0.832	0.608–1.140	0.253
Setting	1L	84	Ref			84	Ref		
	2L	126	1.148	0.812–1.624	0.435	126	1.227	0.892–1.688	0.209
Neoadjuvant chemotherapy	No	190	Ref			190	Ref		
	Yes	20	0.873	0.483–1.578	0.652	20	0.810	0.468–1.403	0.453
Radiochemotherapy	No	188	Ref			188	Ref		
	Yes	22	0.812	0.449–1.468	0.491	22	0.771	0.446–1.334	0.353
Radiotherapy	No	131	Ref			131	Ref		
	Yes	77	1.210	0.857–1.708	0.278	77	1.237	0.897–1.707	0.194
Radical surgery	No	118	Ref			118	Ref		
	Yes	92	0.659	0.468-0.0929	**0.017**	92	0.768	0.562–1.051	0.099
ECOG PS	0	117	Ref			117	Ref		
	1 +	82	1.902	1.347–2.686	** < 0.001**	82	1.562	1.133–2.154	**0.007**
Liver metastasis	No	176	Ref			176	Ref		
	Yes	33	2.888	1.907–4.373	** < 0.001**	33	2.588	1.725–3.883	** < 0.001**
Visceral metastasis	No	114	Ref			114	Ref		
	Yes	95	1.559	1.117–2.176	**0.009**	95	1.366	1.003–1.859	**0.048**
Bone metastasis	No	161	Ref			161	Ref		
	Yes	49	2.160	1.485–3.141	** < 0.001**	49	2.411	1.688–3.443	** < 0.001**
Lymph node-only metastasis	No	143	Ref			143	Ref		
	Yes	67	0.636	0.439-0.0921	**0.017**	67	0.545	0.384–0.774	** < 0.001**
Hemoglobin level	< 10 g/dl	48	Ref			48	Ref		
	≥ 10 g/dl	147	0.541	0.367–0.800	**0.002**	147	0.644	0.450–0.921	**0.016**
Bellmunt risk factors	0	74	Ref			74	Ref		
	1 +	110	2.933	1.981–4.343	** < 0.001**	110	2.213	1.561–3.137	** < 0.001**
Bellmunt-CRP	0	33	Ref			33	Ref		
	1 +	60	3.567	1.853–6.866	** < 0.001**	60	2.244	1.326–3.795	**0.003**
CRP cut-off	< 30 mg/l	89	Ref			89	Ref		
	≥ 30 mg/l	15	1.876	0.993–3.543	0.052	15	1.627	0.895–2.956	0.110
NLR cut-off	< 5	109	Ref			109	Ref		
	≥ 5	47	1.883	1.230–2.882	**0.004**	47	2.170	1.476–3.191	** < 0.001**
LDH cutoff	< 250 U/L	68	Ref			68	Ref		
	≥ 250 U/L	71	1.304	0.864–1.967	0.206	71	1.221	0.841–1.773	0.293
Albumin cut-off	< 35 g/l	27	Ref			27	Ref		
	≥ 35 g/l	102	0.418	0.256–0.683	** < 0.001**	102	0.549	0.344–0.878	**0.012**
eGFR cut-off	< 40 ml/min	27	Ref			27	Ref		
	≥ 40 ml/min	111	0.514	0.309–0.857	**0.011**	111	0.756	0.471–1.215	0.249

Significant values are in bold.

**Figure 1:**
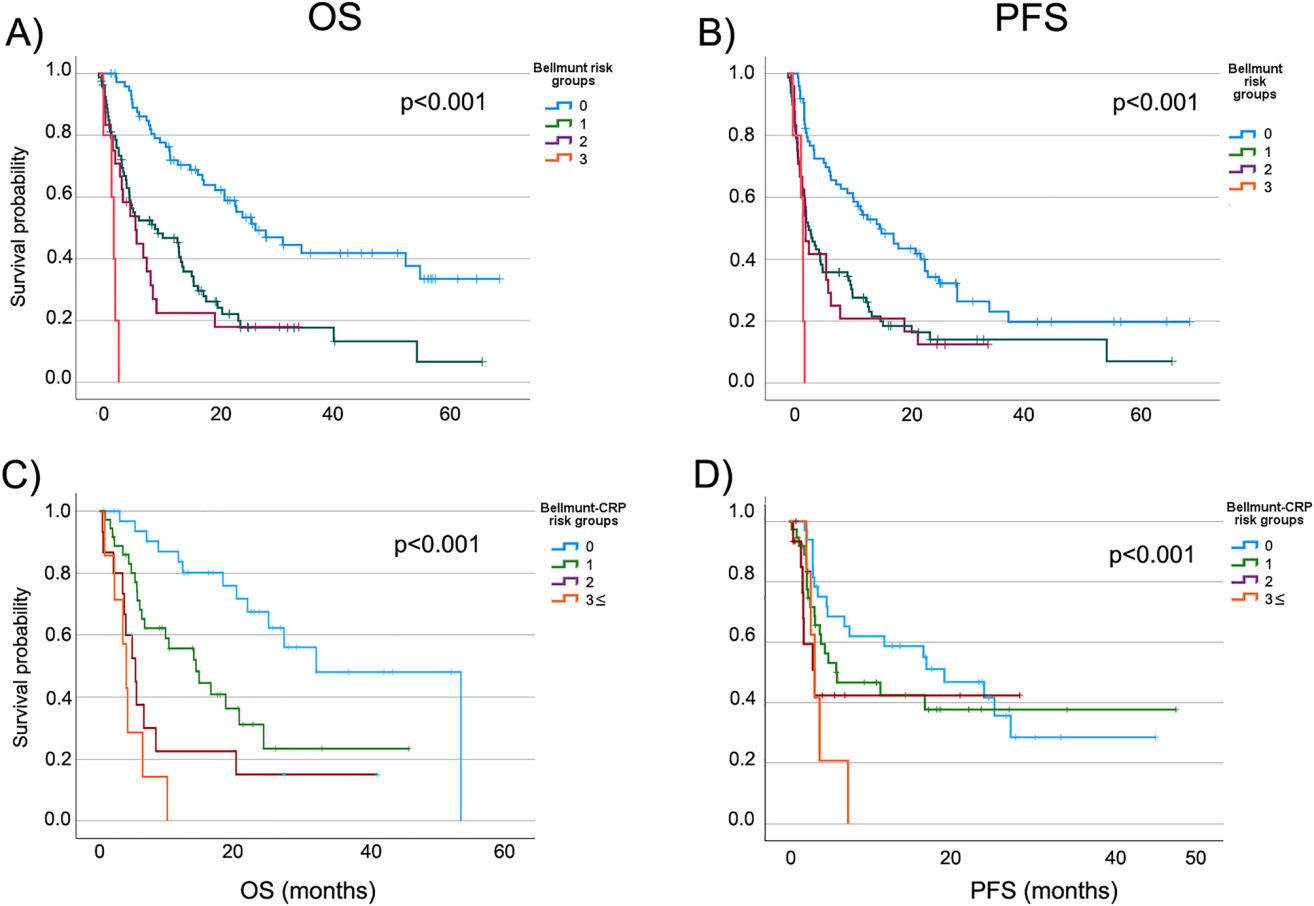
Overall survival (OS) (left) and progression-free survival (PFS) (right) for patients grouped according to Bellmunt risk factors (**A**,**B**) and Bellmunt-CRP risk factors (**C**,**D**). Median OS for Bellmunt risk score groups (**A**) were as follows 26.9, 9.6, 6.3 and 2.6 months. Median PFS for Bellmunt risk score groups (**B**) were as follows 15.6, 3.6, 3.0 and 2.5 months. Median OS for Bellmunt-CRP risk score groups (**C**) were as follows 31.5, 14.1, 5.2, 3.9 months. Median PFS for Bellmunt-CRP risk score groups (**D**) were as follows 18.1, 5.6, 3.0 and 3.0 months.

In Cox regression analysis examining PFS age (> 68 years), LN only metastases, hemoglobin and albumin levels above the cutoff values were associated with improved PFS, while the presence of liver, visceral or bone metastases, ECOG-PS, Bellmunt and Bellmunt-CRP risk groups (1 +), and high NLR value were associated with worse PFS (Fig. 1A–D; Table 3). Univariate Cox regression analyses were also performed separately for first-, and second-line treatment groups. The detailed results are shown in Suppl. Tables 1 and 2.

**Table 3:** Association between the response, disease control and different clinicopathological variables (Chi-squared test).

Variables		All patients n (%)	Disease control			Response		
			PD	SD/PR/CR	p	PD/SD	PR/CR	p
			n (%)	n (%)		n (%)	n (%)	
Sex	Male	124 (69)	46 (37)	78 (63)	0.675	77 (60)	47 (40)	0.553
	Female	57 (31)	23 (40)	34 (60)		38 (67)	19 (33)	
Age at ICI	≤ 68 years	74 (41)	37 (50)	37 (50)	**0.006**	57 (77)	17 (23)	**0.002**
	> 68 years	107 (59)	32 (30)	75 (70)		58 (54)	49 (46)	
Drug	Atezolizumab	64 (35)	28 (44)	36 (56)	0.249	41 (64)	23 (36)	0.913
	Pembrolizumab	117 (65)	41 (35)	76 (65)		74 (63)	43 (37)	
ICI setting	1L	73 (40)	25 (34)	48 (66)	0.378	49 (67)	24 (33)	0.410
	2L	108 (60)	44 (41)	64 (59)		66 (61)	42 (39)	
Tumor site	BC	148 (86)	58 (39)	90 (61)	0.875	97 (66)	51 (34)	0.282
	UTUC	24 (14)	9 (38)	15 (62)		13 (54)	11 (46)	
Radical surgery	Yes	84 (46)	29 (35)	55 (65)	0.354	48 (57)	36 (43)	0.096
	No	97 (54)	40 (41)	57 (59)		67 (69)	30 (31)	
Radiochemotherapy	No	160 (88)	62 (39)	98 (63)	0.631	100 (63)	60 (37)	0.424
	Yes	21 (12)	7 (33)	14 (67)		15 (71)	6 (29)	
Bellmunt risk factors	0	72 (43)	17(23)	55 (77)	**0.002**	35 (48)	37 (52)	**0.001**
	1 +	95 (57)	45 (47)	50 (53)		70 (74)	25 (26)	
Bellmunt-CRP	0	33 (39)	9 (27)	24 (73)	**0.016**	16 (49)	17 (51)	**0.007**
	1 +	52 (61)	28 (54)	24 (46)		40 (77)	12 (23)	
ECOG PS	0	104 (60)	31 (30)	73 (70)	**0.016**	57 (55)	47 (45)	**0.006**
	1 +	69 (40)	33 (48)	36 (52)		52 (75)	17 (25)	
Liver metastasis	No	158 (87)	54 (34)	104 (66)	**0.004**	98 (62)	60 (38)	0.268
	Yes	23 (13)	15 (65)	8 (35)		17 (74)	6 (26)	
Visceral metastasis	No	103 (57)	37 (36)	66 (64)	0.484	69 (67)	34 (33)	0.267
	Yes	78 (43)	32 (41)	46 (59)		46 (59)	32 (41)	
Lymph node-only mets	No	121 (67)	55 (45)	66 (55)	**0.004**	80 (66)	41 (34)	0.306
	Yes	60 (33)	14 (23)	46 (77)		35 (58)	25 (42)	
Bone metastasis	No	140 (77)	41 (29)	99 (71)	** < 0.001**	82 (59)	58 (41)	**0.010**
	Yes	41 (23)	28 (68)	13 (32)		33 (80)	8 (20)	
CRP	< 30 mg/l	80 (86)	33 (41)	47 (59)	0.060	51 (64)	29 (36)	0.139
	≥ 30 mg/l	13 (14)	9 (69)	4 (31)		11 (85)	2 (15)	
LDH	< 250 U/L	62 (49)	22 (35)	40 (65)	0.381	38 (61)	24 (39)	0.451
	≥ 250 U/L	65 (51)	28 (43)	37 (57)		44 (68)	21 (32)	
NLR	< 5	102 (70)	31 (30)	71 (70)	**0.018**	55 (54)	47 (46)	**0.005**
	≥ 5	43 (30)	22 (51)	21 (49)		34 (79)	9 (21)	
Hemoglobin	< 10 g/dl	41 (23)	20 (49)	21 (51)	0.114	31 (76)	10 (24)	0.064
	≥ 10 g/dl	134 (77)	47 (35)	87 (65)		80 (60)	54 (40)	
Albumin	< 35 g/l	23 (19)	12 (52)	11 (48)	**0.039**	21 (91)	2 (9)	**0.001**
	≥ 35 g/l	98 (81)	29 (30)	69 (70)		53 (54)	45 (46)	
eGFR	< 40 ml/min	25 (20)	12 (48)	13 (52)	0.275	18 (72)	7 (28)	0.164
	≥ 40 ml/min	97 (80)	35 (36)	62 (64)		55 (57)	42 (43)3)	

Significant values are in bold.

Multivariate models included all the variables that showed significant associations with survival and were available for at least 85% of the cases. In multivariable analysis ECOG-PS, presence of visceral or bone metastases, and hemoglobin levels (> 10 g/dl) were found to be independent risk factors for OS. The presence of one or more Bellmunt risk factors proved to be an independent prognostic factor for shorter OS and PFS as well (Supplementary Table 3).

### Factors associated with radiographic response or disease control

Information on therapy response during ICI treatment was available for a subset of patients (n = 181). Age at ICI initiation, ECOG-PS, presence of bone metastases, and Bellmunt risk factors as well as NLR and albumin levels showed significant associations with response and disease control (Table 3).

## Discussion

The introduction of ICIs has significantly improved the treatment paradigm for mUC. Prospective clinical trials have shown that these agents can provide durable therapeutic effects and prolong survival, however, only in a rather small subgroup of patients^2,6–8,15,16^. Clinical trials include highly selected patient cohorts, which may not broadly represent real-world outcomes^17^. Therefore, real-world data received substantial attention in recent years, as it enables us to understand the practicality of medical interventions in a wider and more representative patient population^18^.

The multicentric nature of this study allowed the inclusion of a wide range of patients from the real clinical practice. Patient characteristics (age, sex, location of the primary tumor) of our cohort was comparable to those of the respective randomized clinical trials (KEYNOTE-045, KEYNOTE-052, IMvigor211 and IMvigor210), with only few exceptions (Table 4).

**Table 4:** Comparison of present real-world cohort with patient cohorts in the corresponding RCTs.

	Pembrolizumab								Atezolizumab									
	First-line				Second-line				First-line				Second-line					
	Real-world cohort		KEYNOTE-052		Real-world cohort		KEYNOTE-045		Real-world cohort		IMvigor210 Cohort 1		Real-world cohort		IMvigor210 Cohort 2		IMvigor211	
	n = 66	%	n = 370	%	n = 68	%	n = 270	%	n = 18	%	n = 119	%	n = 58	%	n = 310	%	n = 467	%
Age at ICI initiation, years median	72		74		68		67		72		73		68		66		67	
Sex																		
Male, no (%)	41	62.1	286	77.3	55	80.9	200	74.1	10	55.6	96	80.7	40	69.0	241	77.7	110	23.6
Female, no (%)	25	37.9	84	22.7	13	19.1	70	25.9	8	44.4	23	19.3	18	31.0	69	22.3	357	76.4
Location of primary tumor																		
Upper urinary tract, no (%)	6	9.1	69	18.6	9	13.2	38	14.1	0	0.0	33	27.7	11	19.0	65	21.0	126	27.0
Bladder, no (%)	58	87.9	300	81.1	54	79.4	232	85.9	16	88.9	85	71.4	44	75.9	230	74.2	324	69.4
Boths, no (%)	2	3.0	0	0.0	5	7.4	0	0.0	2	11.1			3	5.2				
ECOG-PS at ICI initiation																		
0, no(%)	36	54.5	80	21.6	39	57.4	119	44.1	3	16.7			39	67.2	117	37.7	218	46.7
1, no (%)	19	28.8	133	35.9	15	22.1	143	53.0	9	50.0			15	25.9	193	62.3	249	53.3
2, no (%)	9	13.6	156	42.2	6	8.8	2	0.7	6	33.3	24	20.2	1	1.7				
3, no (%)	0	0.0%	1	0.3	1	1.5	0	0.0	0	0.0			1	1.7				
Unknown, no (%)	2	3.0	0	0.0	7	10.3	6	2.2	0	0.0			2	3.4				
Liver metastasis, no (%)	7	10.6	78	21.1	14	20.6	91	33.7	2	11.1%	25	21.0	10	17.2	96	31.0	138	29.6
ORR, no (%)	21	36.8	106	28.6	22	36.7	57	21.1	3	18.8	27	22.7	20	41.7	45	14.5	62	13.3
DCR, % (95% CI)	40	70.2	173	46.8	36	60.0	104	38.5	8	50.0	56	47.1	28	58.3	104	33.5	154	33.0
Best overall response																		
Complete response, no (%)	8	12.1	33	8.9	4	5.9	25	9.3	0	0.0	11	9.2	1	1.7	15	4.8	16	3.4
Partial response, no (%)	13	19.7	73	19.7	18	26.5	32	11.9	3	16.7	16	13.4	19	32.8	30	9.7	46	9.9
Stable disease, no (%)	19	28.8	67	18.1	14	20.6	47	17.4	5	27.8	29	24.4	8	13.8	59	19.0	92	19.7
Progressive disease, no (%)	17	25.8	157	42.4	24	35.3	131	48.5	8	44.4	43	36.1	20	34.5	159	51.3	240	51.4
Not available, no (%)	9	13.6	40	10.8	8	11.8	35	13.0	2	11.1	20	16.8	10	17.2	47	15.2	73	15.6
PFS, months, median	8.1		2.0		3.6		2.1		2.0		2.7		6.4		2.1		2.1	
Death, no (%)	37	56.1	239	64.6	46	67.6	208	77.0	13	72.2	59	49.6	44	75.9	193	62.3	324	69.4
OS, months, median	15.6		11.3		8.8		10.3		5.6		15.9		17.0		7.9		11.1	

Interestingly, our real-life cohort included a lower rate of liver metastasis, which is an unexpected finding considering that investigators often tend to select more fit patients for inclusion in RCTs. A similar phenomenon was observed by Omland et al*.* when comparing a real-life cohort of Danish UC patients treated with pembrolizumab to cohorts from clinical trials^19^. The presence of liver metastasis is a well-known risk factor of OS. In line with our findings, a meta-analysis has reported a significant association between the presence of visceral or liver metastasis and worse OS in the pembrolizumab-treated UC patient cohort^20^. In addition, recent findings have shown that radical surgery of the primary tumor does not confer with OS benefits in patients with liver metastasis^21^. Due to the shorter life expectancy of these patients, they frequently do not receive any systematic treatment. This could serve as a plausible explanation for the underrepresentation of patients with liver metastasis in our real-life cohort. Another explanation might be that oncologists in real clinical practice may prefer chemotherapy for these patients or inclusion in clinical trials such as those involving antibody–drug conjugates, which were actively recruiting patients during the timeframe of this retrospective cohort study.

The ORRs observed in our study were similar to those reported in the RCTs, with exception of second-line atezolizumab treatment. In this setting, the ORR of the real-life cohort (41.7%) was more than 25% higher compared to the respective clinical trials (14.5% and 13.3% in the IMvigor210/cohort 2 and IMvigor211, respectively). Similarly, a real-life study by Tural et al*.* investigating an atezolizumab-treated UC population reported also a higher ORR rate (28.7%) for the second-line setting^22^.

The median OS time for our whole cohort was 13.6 months. When dividing the patients according to the applied drugs and settings; the worst OS could be observed in the first-line atezolizumab-treated patients’ group, although the case numbers in this subgroup are low. There was a relevant OS difference in the second-line atezolizumab-treated groups between the real-life (17.0 months) and RCT cohorts (7.9 and 11.1 months in the IMvigor210/cohort 2 and IMvigor211, respectively). According to this observation, also other real-life studies found a significantly longer survival for this subgroup comparing to the respective RCTs, suggesting an even higher benefit for this treatment setting in real-world conditions as observed in former clinical trials^22–24^.

In this study, impaired ECOG-PS, the presence of visceral, liver, or bone metastases, and baseline hemoglobin levels below 10 g/dl were found to be independent prognostic factors for OS. In addition, the presence of one or more Bellmunt risk factors proved to be an independent prognostic factor for shorter OS.

ECOG-PS is a validated prognostic parameter in oncology outpatient settings. It has previously been demonstrated as an independent prognostic factor for OS in patients with advanced UC who are treated with platinum-containing regimens^14^. In line with our present results, Yanagisawa et al*.* reported worse OS for UC patients with poor ECOG-PS despite pembrolizumab treatment^20^. In a retrospective cohort study, Khaki et al*.* investigated real-life patient data and reported shorter OS for patients with ECOG-PS ≥ 2, particularly in the first-line setting^25^. Similar results have been reported by Tural et al*.* in the second-line setting for a real-life cohort^22^.

The Bellmunt risk score is a further well-established prognostic factor in the second-line treatment setting UC^6^. While initially developed based on a chemotherapy cohort, its simplicity led to its utilization for risk stratification of ICI-treated patients. In order to enhance discrimination of the Bellmunt risk score in ICI-treated patients with UC, an enhanced version known as theBellmunt-CRP score has been recently established by Abuhelwa et al*.*^26^. In this study, we assessed the performance of the Bellmunt-CRP risk score for the first time on real-life data and could validate its improved ability to discriminate high-risk patients. However, this association was not found to be statistically significant in the first-line cohort.

Regarding hematological biomarkers, elevated pre-treatment levels of NLR and low albumin levels were found to be significantly associated with worse OS, PFS, and response to ICI therapy in various cancers^27,28^. Nassar et al*.* showed an association between high NLR and lack of clinical benefit to ICIs in UC patients^29^. The cause of the elevated ratio can be either higher neutrophil abundance or lower levels of lymphocytes. On one hand, neutrophil infiltration in cancer tissue can contribute to a pro-tumor microenvironment, as they are able to secrete immunosuppressive mediators and angiogenic factors (e.g. reactive oxygen species, vascular endothelial growth factor and matrix metalloproteinase 9), which can promote tumor growth and progression^30^. On the other hand, low levels of circulating lymphocytes may correlate with a decreased number of tumor-infiltrating lymphocytes and thus a reduced anti-tumor T-cell response^31^. The here presented results confirm that NLR is a suitable candidate for a cost-effective and widely accessible biomarker.

Our study has several limitations. First, the retrospective study design limits the availability of the collected data. In addition, detailed information about side effects is missing. Second, our cohort was heterogeneous regarding the line of therapy, and previous chemotherapy regimens. Third, real-life circumstances did not allow a strict timing of imaging analyses and there was no opportunity for a central evaluation of CT/MRI scans. Thus, treatment response was determined by the data collector, based on clinical reports, which may affect the interpretation of results. Fourth, PD-L1 immunohistostaining was available only for a limited number of patients with heterogeneous assay and evaluation methods, which did not allow us the performance of a statistically valid evaluation. Therefore, the therapy predictive value of tissue PD-L1 expression could not be assessed in this study.

In conclusion, in this multicentre study, we demonstrated that atezolizumab and pembrolizumab are effective treatment options for a broad range of mUC patients regardless of treatment line. Second-line atezolizumab provided higher response and OS rates compared to the respective RCTs. As the treatment landscape for mUC is continuously expanding, easily accessible markers like clinicopathological and laboratory parameters are essential for better therapeutic decision-making. Our results demonstrated the prognostic value of a series of risk factors such as ECOG-PS, liver, visceral, bone or LN only metastases, NLR, hemoglobin, albumin, eGFR levels and we were able to confirm that the prognostic value of Bellmunt risk scores can further be increase when adding CRP to the model. Based on these, our study contributes to the current body of evidence by reporting valuable real-life experiences and pointing out some important similarities and differences to the approval studies. These data will provide important input to larger meta-analyses and may contribute to clinical conclusions on high clinical evidence level. Finally, relevant real-world studies will help to place ICI therapy in the evolving treatment context of mUC.

## Methods

### Patients and data collection

**Figure 2:**
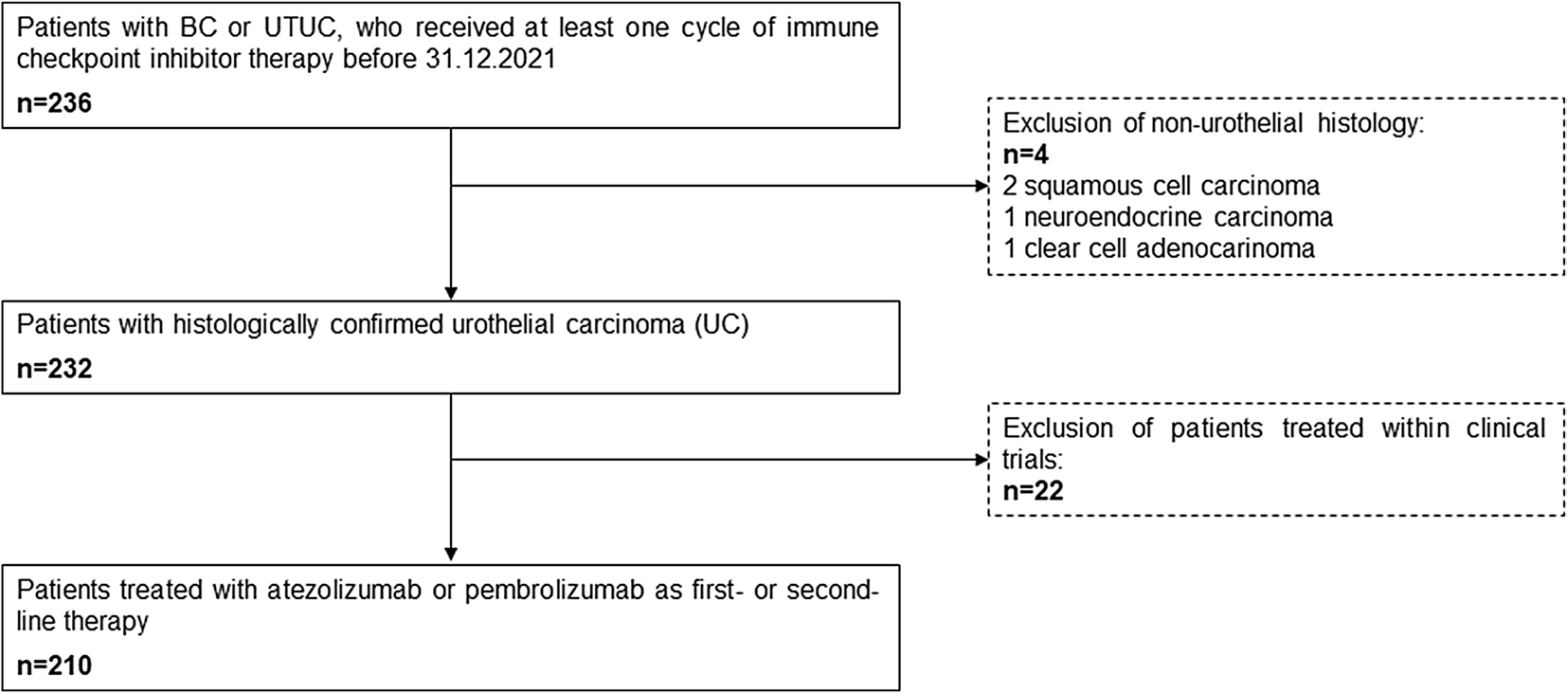
Flow chart of the cohort selection (*BC* bladder cancer, *UTUC* upper urinary tract cancer).

Eligible patients for inclusion were adults (≥ 18 years) with a confirmed diagnosis of advanced or metastatic urothelial tract malignancy, who received at least one cycle of ICI therapy (pembrolizumab or atezolizumab) as first- or second-line treatment between 01/2017 and 12/2021. Patients with non-urothelial histology and those who were treated within clinical trials were excluded (Fig. 2). Patients’ data were obtained from medical records at 6 urooncology centers (Semmelweis University; National Institute of Oncology; University Hospital Duesseldorf; University of Duisburg-Essen; University of Szeged; Borsod-Abaúj-Zemplén County Hospital). Clinicopathological, laboratory, and outcome data were collected. Previously reported cutoff values were applied to the laboratory parameters. According to the original and the enhanced Bellmunt risk score 10 g/dl, 30 mg/l, and 5 were used as the cutoffs for hemoglobin, C-reactive protein (CRP), and neutrophil–lymphocyte-ratio (NLR), respectively^14,26^. In addition, the lower limit of the normal range was employed as the cutoff value for albumin (35 g/l), and the upper limit of the normal range as the cutoff for LDH (250 U/l). For estimated glomerular filtration rate (eGFR), we applied the 40 ml/min as the cut-off value. The follow-up cutoff was at 08/2022. The study conformed to the Declaration of Helsinki and the institutional ethics committee (Semmelweis Egyetem Regonális, Intézményi Tudományos és Kutatásetikai Bizottsága and Ethik Kommission Medizinische Fakultät der Universität Duisburg-Essen) approved the study protocol (SE RKEB 125/2019, 15–6400-BO, 2021–1548). Informed consent was obtained from all participants.

### Outcomes

OS was the primary endpoint, while the secondary endpoints included progression-free survival (PFS), ORR, disease control rate (DCR), and duration of response (DOR). OS was defined as the period from ICI therapy initiation to the date of death, whereas PFS referred to the time from the first ICI treatment to the date of disease progression (radiographic or clinical) or death. Patients who did not die during the observation period were censored at the last follow-up. Responses were assessed using computed tomography (CT) scans according to institutional standard in patients with at least one follow-up scan during treatment. CT scans were evaluated by local radiologists at each center as part of the daily routine, following the Response Evaluation Criteria in Solid Tumors (RECIST) guideline version 1.1. Treatment response was determined by the data collector based on radiographic studies and clinic notes. ORR was defined as the percentage of patients who achieved a PR or CR to the treatment. DCR included patients with CR, PR, or stable disease (SD). Time-to-response referred to the duration from the first cycle until the first documented response. DOR was defined as the time from the first documented radiological response to disease progression or death.

### Statistical analysis

Descriptive statistics included median and range for continuous variables and numbers and percentages for categorical variables. All time-to-event data (OS, PFS, DOR) are summarized using Kaplan–Meier estimates, and medians are reported with corresponding 95% confidence intervals (CI). The median and range of time-to-response are reported for patients with CR and PR. Cox proportional hazard models were used to assess differences in hazard ratios (HR) between groups according to the risk factors and stratified two-sided log-rank test was used to assess differences in OS. Chi-square test was used to determine the association between response (CR/PR) or disease control (CR/PR/SD) and different clinicopathological variables. A p-value of < 0.05 was considered significant. All statistical analyses were performed with SPSS version 27.0 (IBM, Armonk, N.Y., USA).

### Supplementary Information

Supplementary Tables.

## Data Availability

The data sets generated during and/or analyzed during the current study are available from the corresponding author on reasonable request.
