# Supplementary material for: Efficacy of immune checkpoint inhibitor therapy for advanced urothelial carcinoma in real-life clinical practice: results of a multicentric, retrospective study
Source: Sci Rep. 2023 Oct 13;13:17378. doi: 10.1038/s41598-023-44103-9 (PMC10575904; doi:10.1038/s41598-023-44103-9)
Supplement: Supplementary file 1 — Supplementary Tables. [file 41598_2023_44103_MOESM1_ESM.pdf]

Efficacy of immune checkpoint inhibitor therapy for advanced urothelial carcinoma in real-life clinical practice: Results of a multicentric, retrospective study

Melinda Váradi[1], Orsolya Horváth[2], Orsolya Módos[1], Tamás Fazekas[1], Camilla M. Grunewald [3], Günter Niegisch[3], Ulrich Krafft[4], Viktor Grünwald[4], Boris Hadaschik[4], Csilla Olah[4], Anikó Maráz[5], Andrea Furka[6,7], Miklós Szűcs[1], Péter Nyirády[1], Tibor Szarvas[1,4*]

[1]Department of Urology, Semmelweis University, Budapest, Hungary
[2]Department of Genitourinary Medical Oncology and Pharmacology, National Institute of Oncology, Budapest
[3]Department of Urology, Medical Faculty and University Hospital Duesseldorf, Heinrich-Heine-University Duesseldorf, Duesseldorf, Germany
[4]Department of Urology, University of Duisburg-Essen, Essen, Germany
[5]Department of Oncotherapy, University of Szeged, Szeged, Hungary
[6]Department of Oncology, Faculty of Medicine, University of Debrecen, Debrecen, Hungary
[7]Department of Clinical Radiology, Institute of Practical Methodology and Diagnostics, Faculty of Health Care, University of Miskolc, Miskolc, Hungary

***Corresponding author:** Tibor Szarvas, PhD
Department of Urology, Semmelweis University, Üllői út 78/B. 1082 Budapest, Hungary
Department of Urology, University Duisburg-Essen, Essen, Hufelandstr 55, 45147, Essen, Germany
Tel.: +36-1-210-0280
Fax.: +36-1-210-0305
E-mail: szarvas.tibor@semmelweis.hu; tibor.szarvas@uk-essen.de

Supplementary Table 1. Univariate Cox regression analysis for the first-line cohort

**Variables**		**Overall survival**				**Progression-free survival**			
		**n**	**HR**	**95% CI**	**p**	**n**	**HR**	**95% CI**	**p**
Age at ICI initiation	≤ 68	26	ref.			26	ref.		
	> 68	58	0.857	0.476-1.540	0.605	58	0.755	0.438-1.303	0.313
Sex	Male	51	ref.			51	ref.		
	Female	33	1.008	0.568-1.787	0.979	33	0.949	0.561-1.606	0.845
ICI drug	Atezolizumab	18	ref.			18	ref.		
	Pembrolizumab	66	0.554	0.293-1.049	0.070	66	0.577	0.320-1.041	0.068
Tumor site	Bladder	74	ref.			74	ref.		
	Upper urinary tract	6	1.509	0.460-4.949	0.497	6	1.686	0.667-4.266	0.270
Neoadjuvant chemotherapy	No	77	ref.			77	ref.		
	Yes	7	1.971	0.824-4.714	0.127	7	1.731	0.738-4.063	0.207
Radiochemotherapy	No	66	ref.			66	ref.		
	Yes	18	0.561	0.263-1.198	0.135	18	0.628	0.318-1.239	0.179
Radiotherapy	No	45	ref.			45	ref.		
	Yes	38	1.122	0.638-1.973	0.689	38	1.066	0.637-1.784	0.807
Radical surgery	No	55	ref.			55	ref.		
	Yes	29	0.562	0.298-1.059	0.075	29	0.771	0.446-1.331	0.350
ECOG PS	0	39	ref.			39	ref.		
	1+	43	1.388	0.787-2.449	0.257	43	1.359	0.812-2.274	0.244
Liver metastasis	No	74	ref.			74	ref.		
	Yes	9	1.720	0.766-3.864	0.189	9	1.529	0.721-3.240	0.268
Visceral metastasis	No	51	ref.			51	ref.		
	Yes	32	1.203	0.683-2.119	0.521	32	1.217	0.724-2.048	0.458
Bone metastasis	No	75	ref.			75	ref.		
	Yes	9	1.469	0.624-3.459	0.379	9	3.094	1.436-6.666	**0.004**
Lymph node-only metastasis	No	55	ref.			55	ref.		
	Yes	29	1.094	0.613-1.951	0.761	29	0.686	0.394-1.194	0.182
Hemoglobin level	< 10 g/dl	17	ref.			17	ref.		
	≥ 10 g/dl	56	0.805	0.393-1.648	0.552	56	0.694	0.374-1.289	0.247
Bellmunt risk factors	0	19	ref.			19	ref.		
	1+	52	1.634	0.780-3.426	0.193	52	1.845	0.945-3.603	0.073
Bellmunt-CRP	0	10	ref.			10	ref.		
	1+	29	1.972	0.566-6.872	0.287	29	2.013	0.755-5.368	0.162
CRP cutoff	< 30 mg/l	37	ref.			37	ref.		
	≥ 30 mg/l	4	1.029	0.230-4.606	0.970	4	2.079	0.619-6.978	0.236
NLR cutoff	< 5	34	ref.			34	ref.		
	≥ 5	19	1.923	0.905-4.086	0.089	19	2.909	1.460-5.794	**0.002**
LDH cutoff	< 250 U/L	31	ref.			31	ref.		
	≥ 250 U/L	23	1.405	0.683-2.890	0.356	23	1.422	0.750-2.694	0.281
Albumin cutoff	< 35 g/l	6	ref.			6	ref.		
	≥ 35 g/l	39	1.047	0.307-3.578	0.941	39	0.986	0.341-2.855	0.979
eGFR cutoff	< 40 ml/min	15	ref.			15	ref.		
	≥ 40 ml/min	36	0.850	0.368-1.960	0.702	36	1.111	0.528-2.336	0.781

Supplementary Table 2. Univariate Cox regression analysis for the second-line cohort

**Variables**		**Overall survival**				**Progression-free survival**			
		**n**	**HR**	**95% CI**	**p**	**n**	**HR**	**95% CI**	**p**
Age at ICI initiation	≤ 68	62	ref.			62	ref.		
	> 68	64	0.983	0.649-1.488	0.934	64	0.714	0.484-1.054	0.090
Sex	Male	95	ref.			95	ref.		
	Female	31	1.185	0.735-1.909	0.486	31	1.047	0.661-1.658	0.844
ICI drug	Atezolizumab	58	ref.			58	ref.		
	Pembrolizumab	68	1.099	0.723-1.669	0.659	68	1.047	0.710-1.545	0.816
Tumor site	Bladder	98	ref.			98	ref.		
	Upper urinary tract	20	0.623	0.338-1.148	0.129	20	0.710	0.408-1.234	0.224
Neoadjuvant chemotherapy	No	113	ref.			113	ref.		
	Yes	13	0.548	0.239-1.256	0.155	13	0.527	0.255-1.088	0.083
Radiochemotherapy	No	122	ref.			122	ref.		
	Yes	4	3.994	1.430-11.152	**0.008**	4	2.242	0.813-6.185	0.119
Radiotherapy	No	86	ref.			86	ref.		
	Yes	39	1.341	0.863-2.086	0.192	39	1.500	0.985-2.283	0.059
Radical surgery	No	63	ref.			63	ref.		
	Yes	63	0.674	0.444-1.025	0.065	63	0.691	0.467-1.021	0.064
ECOG PS	0	78	ref.			78	ref.		
	1+	39	2.715	1.725-4.274	**<0.001**	39	1.941	1.265-2.980	**0.002**
Liver metastasis	No	102	ref.			102	ref.		
	Yes	24	3.624	2.213-5.937	**<0.001**	24	3.834	2.302-6.384	**<0.001**
Visceral metastasis	No	63	ref.			63	ref.		
	Yes	63	1.780	1.172-2.703	**0.007**	63	1.455	0.986-2.147	0.059
Bone metastasis	No	86	ref.			86	ref.		
	Yes	40	2.333	1.499-3.630	**<0.001**	40	2.187	1.440-3.323	**<0.001**
Lymph node-only metastasis	No	88	ref.			88	ref.		
	Yes	38	0.459	0.280-0.750	**0.002**	38	0.469	0.289-0.740	**0.001**
Hemoglobin cutoff	< 10 g/dl	31	ref.			31	ref.		
	≥ 10 g/dl	91	0.437	0.273-0.697	**0.001**	91	0.619	0.399-0.962	**0.033**
Bellmunt risk factors	0	55	ref.			55	ref.		
	1+	58	4.672	2.872-7.601	**<0.001**	58	2.924	1.897-4.508	**<0.001**
Bellmunt-CRP	0	23	ref.			23	ref.		
	1+	31	5.639	2.580-12.326	**<0.001**	31	2.823	1.486-5.362	**0.002**
CRP cutoff	< 30 mg/l	52	ref.			52	ref.		
	≥ 30 mg/l	11	2.057	0.998-4.240	0.051	11	1.402	0.700-2.805	0.340
NLR cutoff	< 5	75	ref.			75	ref.		
	≥ 5	28	1.802	1.073-3.026	**0.026**	28	1.851	1.155-2.965	**0.010**
LDH cutoff	< 250 U/L	37	ref.			37	ref.		
	≥ 250 U/L	48	1.234	0.744-2.045	0.415	48	1.088	0.685-1.729	0.721
Albumin cutoff	< 35 g/l	21	ref.			21	ref.		
	≥ 35 g/l	63	0.316	0.180-0.556	**<0.001**	63	0.436	0.254-0.747	**0.003**
eGFR cutoff	< 40 ml/min	12	ref.			12	ref.		
	≥ 40 ml/min	75	0.322	0.168-0.619	**0.001**	75	0.493	0.263-0.925	**0.028**

Supplementary Table 3. Multivariable Cox regression analysis

Varibles		Overall survival				Progression-free survival			
		**n**	**HR**	**95% CI**	**p**	**n**	**HR**	**95% CI**	**p**
**Model 1**									
ECOG PS	0	108	ref.			108	ref.		
	1+	76	1.909	1.304-2.795	**<0.001**	76	1.454	1.020-2.074	**0.039**
Visceral metastasis	No	104	ref.			104	ref.		
	Yes	80	1.812	1.085-3.025	**0.023**	80	1.109	0.725-1.697	0.633
Radical surgery	No	99	ref.			99	ref.		
	Yes	85	0.721	0.491-1.058	0.095	85	0.823	0.582-1.164	0.271
Bone metastasis	No	140	ref.			140	ref.		
	Yes	44	2.327	1.446-3.743	**<0.001**	44	2.017	1.315-3.095	**0.001**
Lymph node-only metastasis	No	123	ref.			123	ref.		
	Yes	61	1.564	0.858-2.854	0.145	61	0.798	0.479-1.328	0.385
Hemoglobin level	< 10 g	46	ref.			46	ref.		
	≥ 10 g	138	0.651	0.425-0.997	**0.048**	138	0.750	0.507-1.110	0.151
**Model 2**									
Bellmunt risk factors	0	74	ref.			74	ref.		
	1+	110	2.638	1.762-3.949	**<0.001**	110	2.017	1.408-2.891	**<0.001**
Radical surgery	No	99	ref.			99	ref.		
	Yes	85	0.741	0.509-1.080	0.119	85	0.888	0.630-1.252	0.499
Bone metastasis	No	140	ref.			140	ref.		
	Yes	44	1.978	1.278-3.060	**0.002**	44	2.001	1.331-3.008	**<0.001**
Lymph node-only metastasis	No	123	ref.			123	ref.		
	Yes	61	1.051	0.683-1.618	0.821	61	0.759	0.509-1.132	0.176

Supplementary Table 4. Association between the response, disease control and different clinicopathological variables in first-line treatment setting (Chi-squared test)

Variables		All patients n (%)	Disease control			Response		
			PD n (%)	SD/PR/CR n (%)	p	PD/SD n (%)	PR/CR n (%)	p
Sex	Male	44 (60)	14 (32)	30 (68)	0.590	27 (61)	17 (39)	0.197
	Female	29 (40)	11 (38)	18 (62)		22 (76)	7 (24)	
Age at ICI	≤68 years	21 (29)	9 (43)	12 (57)	0.325	17 (81)	4 (19)	0.110
	>68 years	52 (71)	16 (31)	36 (69)		32 (62)	20 (38)	
Drug	Atezolizumab	16 (22)	8 (50)	8 (50)	0.133	13 (81)	3 (19)	0.173
	Pembrolizumab	57 (78)	17 (30)	40 (70)		36 (63)	21 (37)	
Tumor site	BC	63 (91)	21 (33)	42 (67)	0.413	42 (67)	21 (33)	0.403
	UTUC	6 (9)	3 (50)	3 (50)		5 (83)	1 (17)	
Radical surgery	Yes	29 (40)	10 (34)	19 (66)	0.972	17 (59)	12 (41)	0.209
	No	44 (60)	15 (34)	29 (66)		32 (73)	12 (27)	
Radiochemotherapy	No	56 (77)	19 (34)	37 (66)	0.917	37 (66)	19 (34)	0.728
	Yes	17 (23)	6 (35)	11 (65)		12 (71)	5 (29)	
Bellmunt risk factors	0	19 (29)	4 (21)	15 (79)	0.100	11 (58)	8 (42)	0.185
	1+	47 (71)	20 (43)	27 (57)		35 (74)	12 (26)	
Bellmunt-CRP	0	10 (28)	3 (30)	7 (70)	0.379	6 (60)	4 (40)	0.310
	1+	26 (72)	12 (46)	14 (54)		20 (77)	6 (23)	
ECOG PS	0	32 (45)	8 (25)	24 (75)	0.103	18 (56)	14 (44)	**0.035**
	1+	39 (55)	17 (44)	22 (56)		31 (79)	8 (21)	
Liver metastasis	No	66 (90)	22 (33)	44 (67)	0.614	46 (70)	20 (30)	0.151
	Yes	7 (10)	3 (43)	4 (57)		3 (43)	4 (57)	
Visceral metastasis	No	46 (63)	14 (30)	32 (70)	0.370	32 (70)	14 (30)	0.526
	Yes	27 (37)	11 (41)	16 (59)		17 (63)	10 (37)	
Lymph node-only metastasis	No	47 (64)	20 (43)	27 (57)	**0.044**	32 (68)	15 (32)	0.055
	Yes	26 (36)	5 (19)	21 (81)		17 (65)	9 (35)	
Bone metastasis	No	65 (89)	18 (28)	47 (72)	**<0.001**	42 (65)	23 (35)	0.194
	Yes	8 (11)	7 (88)	1 (12)		7 (88)	1 (12)	
CRP	<30 mg/l	34 (89)	12 (35)	22 (65)	0.124	22 (65)	12 (35)	0.151
	≥ 30mg/l	4 (11)	3 (75)	1 (25)		4 (100)	0 (0)	
LDH	<250 U/L	28 (56)	8 (29)	20 (71)	0.217	19 (68)	9 (32)	0.981
	≥250 U/L	22 (44)	10 (45)	12 (55)		15 (68)	7 (32)	
NLR	<5	32 (64)	7 (22)	25 (68)	**0.006**	18 (56)	14 (44)	**0.018**
	≥5	18 (36)	11 (61)	7 (39)		16 (89)	2 (11)	
Hemoglobin	<10 g/dl	15 (22)	5 (33)	10 (67)	0.857	11 (73)	4 (27)	0.594
	≥10 g/dl	53 (78)	19 (36)	34 (64)		35 (66)	18 (34)	
Albumin	<35 g/l	6 (14)	2 (33)	4 (67)	0.932	6 (100)	0 (0)	0.058
	≥35 g/l	38 (86)	12 (32)	26 (68)		23 (61)	15 (39)	
eGFR	<40 ml/min	15 (32)	5 (33)	10 (67)	0.632	11 (73)	4 (27)	0.465
	≥40 ml/min	32 (68)	13 (41)	19 (59)		20 (63)	12 (37)	

Supplementary Table 5. Association between the response, disease control and different clinicopathological variables in second-line treatment setting (Chi-squared test)

			**Disease control**			**Response**		
Variables		All patients n (%)	PD n (%)	SD/PR/CR n (%)	p	PD/SD n (%)	PR/CR n (%)	p
Sex	Male	80 (74)	32 (40)	48 (60)	0.791	50 (63)	30 (37)	0.617
	Female	28 (26)	12 (43)	16 (57)		16 (57)	12 (43)	
Age at ICI	≤68 years	53 (49)	28 (53)	25 (47)	**0.012**	40 (75)	13 (25)	**0.003**
	>68 years	55 (51)	16 (29)	39 (71)		26 (47)	29 (53)	
Drug	Atezolizumab	48 (44)	20 (42)	28 (58)	0.861	28 (58)	20 (42)	0.596
	Pembrolizumab	60 (56)	24 (40)	36 (60)		38 (63)	22 (37)	
Tumor site	BC	85 (83)	37 (44)	48 (56)	0.426	55 (65)	30 (35)	0.109
	UTUC	18 (17)	6 (33)	12 (67)		8 (44)	10 (56)	
Radical surgery	Yes	55 (51)	19 (35)	36 (65)	0.182	31 (56)	24 (44)	0.303
	No	53 (49)	25 (47)	28 (53)		35 (66)	18 (34)	
Radiochemotherapy	No	104 (96)	43 (41)	61 (57)	0.514	63 (61)	41 (39)	0.561
	Yes	4 (4)	1 (25)	3 (75)		3 (75)	1 (25)	
Bellmunt risk factors	0	53 (52)	13 (25)	40 (75)	**0.004**	24 (45)	29 (55)	**0.005**
	1+	48 (48)	25 (52)	23 (48)		35 (73)	13 (27)	
Bellmunt-CRP	0	23 (47)	6 (26)	17 (74)	**0.013**	10 (43)	13 (57)	**0.016**
	1+	26 (53)	16 (62)	10 (38)		20 (77)	6 (23)	
ECOG PS	0	72 (71)	23 (32)	49 (68)	**0.043**	39 (54)	33 (46)	0.139
	1+	30 (29)	16 (53)	14 (47)		21 (70)	9 (30)	
Liver metastasis	No	92 (85)	32 (35)	60 (65)	**0.003**	52 (57)	40 (43)	**0.019**
	Yes	16 (15)	12 (75)	4 (25)		14 (88)	2 (12)	
Visceral metastasis	No	57 (53)	23 (40)	34 (60)	0.931	37 (65)	20 (35)	0.392
	Yes	51 (47)	21 (41)	30 (59)		29 (57)	22 (43)	
Lymph node-only metastasis	No	74 (69)	35 (47)	39 (53)	**0.041**	48 (65)	26 (35)	0.238
	Yes	34 (31)	9 (26)	25 (74)		18 (53)	16 (47)	
Bone metastasis	No	75 (69)	23 (31)	52 (69)	**0.001**	40 (53)	35 (47)	0.012
	Yes	33 (31)	21 (64)	12 (36)		26 (79)	7 (21)	
CRP	<30 mg/l	46 (84)	21 (46)	25 (54)	0.249	29 (63)	17 (37)	0.395
	≥ 30mg/l	9 (16)	6 (67)	3 (33)		7 (78)	2 (22)	
LDH	<250 U/L	34 (44)	14 (41)	20 (59)	0.925	19 (56)	15 (44)	0.299
	≥250 U/L	43 (56)	18 (42)	25 (58)		29 (67)	14 (33)	
NLR	<5	80 (76)	24 (30)	46 (70)	0.747	37 (46)	33 (54)	0.096
	≥5	25 (24)	11 (44)	14 (56)		18 (72)	7 (28)	
Hemoglobin	<10 g/dl	26 (24)	15 (58)	11 (42)	**0.036**	20 (77)	6 (23)	**0.052**
	≥10 g/dl	81 (76)	28 (35)	53 (65)		45 (56)	36 (44)	
Albumin	<35 g/l	17 (22)	10 (59)	7 (41)	**0.020**	15 (88)	2 (12)	**0.005**
	≥35 g/l	60 (78)	17 (28)	43 (72)		30 (50)	30 (50)	
eGFR	<40 ml/min	10 (13)	7 (70)	3 (30)	**0.029**	7 (70)	3 (30)	0.338
	≥40 ml/min	65 (87)	22 (34)	43 (66)		35 (54)	30 (46)	
